# Effects of sodium-glucose co-transporter 2 inhibitors on ultrafiltration in patients with peritoneal dialysis: a protocol for a randomized, double-blind, placebo-controlled, crossover trial (EMPOWERED)

**DOI:** 10.1007/s10157-024-02467-w

**Published:** 2024-02-25

**Authors:** Yohei Doi, Maki Shinzawa, Tetsuya Arisato, Hideaki Oka, Ayumi Matsumoto, Harumi Kitamura, Yumi Nakazono, Yoichi Nishiya, Yoshiyasu Ueda, Taro Kamimura, Terumasa Hayashi, Fumiki Yoshihara, Yoshitaka Isaka

**Affiliations:** 1 Department of Nephrology, Osaka University Graduate School of Medicine, 2-2-D11, Yamada-oka, Suita, Osaka 565-0871 Japan; 2 Department of Cardiology, Pulmonology, Hypertension and Nephrology, Ehime University Graduate School of Medicine, Toon, Ehime Japan; 3 Health and Counseling Center, Osaka University, Toyonaka, Osaka Japan; 4 Division of Nephrology and Hypertension, National Cerebral and Cardiovascular Center, Suita, Osaka Japan; 5 Division of Kidney Center, Matsuyama Red Cross Hospital, Matsuyama, Ehime Japan; 6 Department of Clinical Quality Management, Osaka University Hospital, Suita, Osaka Japan; 7 Medicine Division, Nippon Boehringer Ingelheim Co., Ltd., Shinagawa-ku, Tokyo, Japan; 8 Department of Kidney Disease and Hypertension, Osaka General Medical Center, Osaka, Osaka Japan

**Keywords:** SGLT2 inhibitor, Peritoneal dialysis, Ultrafiltration, Volume overload

## Abstract

**Background:**

Volume overload is common and associated with high mortality in patients on peritoneal dialysis (PD). Traditional strategies including diuretics, water/salt restriction, and icodextrin-based solutions cannot always fully correct this condition, necessitating novel alternative strategies. Recent studies confirmed the expression of sodium–glucose cotransporter 2 (SGLT2) in the human peritoneum. Experimental data suggest that SGLT2 inhibitors decrease glucose absorption from the PD solution, thereby increasing the ultrafiltration volume. This trial aims to assess whether SGLT2 inhibitors increase the ultrafiltration volume in patients on PD.

**Methods:**

The EMPOWERED trial (trial registration: jRCTs051230081) is a multicenter, randomized, double-blind, placebo-controlled, crossover trial. Patients with clinically diagnosed chronic heart failure are eligible regardless of the presence of diabetes if they use at least 3 L/day glucose-based PD solutions. Participants will be randomly assigned (1:1) to receive empagliflozin 10 mg once daily and then placebo or vice versa. Each treatment period will last 8 weeks with a 4-week washout period. This study will recruit at least 36 randomized participants. The primary endpoint is the change in the daily ultrafiltration volume from baseline to week 8 in each intervention period. The key secondary endpoints include changes in the biomarkers of drained PD solutions, renal residual function, and anemia-related parameters.

**Conclusions:**

This trial aims to assess the benefit of SGLT2 inhibitors in fluid management with a novel mechanism of action in patients on PD. It will also provide insights into the effects of SGLT2 inhibitors on solute transport across the peritoneal membrane and residual renal function.

**Supplementary Information:**

The online version contains supplementary material available at 10.1007/s10157-024-02467-w.

## Introduction

Peritoneal dialysis (PD) is an established renal replacement therapy, based on the exchange of water and solute between capillary blood and dialysate fluid across the peritoneal membrane, and 11% of patients undergoing dialysis worldwide are on PD [1]. Given the link between volume overload and poor outcomes, encompassing both technical survival and mortality in patients on PD [2], adequate ultrafiltration, relying on the osmotic gradient typically induced by the hypertonic glucose concentration, is a crucial element for successful PD therapy. Ultrafiltration and the volume status are closely interrelated, and in fact, several studies reported that higher ultrafiltration volume was associated with better prognosis in patients on PD [3, 4]. The glucose gradient peaks at the start of dialysis but diminishes as glucose diffuses into blood. In patients with greater peritoneal vascularity in particular, it is difficult to achieve adequate ultrafiltration because the glucose gradient diminishes more rapidly [5]. One typical strategy in this setting is to use a PD solution with higher glucose content to obtain a higher glucose gradient. However, this strategy might be ineffective because of enhanced fluid intake attributable to hyperglycemia, and it can lead to more rapid damage to the peritoneal membrane, resulting in the discontinuation of PD therapy [6].

Recent studies confirmed the expression of sodium–glucose cotransporter 2 (SGLT2) in the human peritoneum [7–9]. Animal studies demonstrated that SGLT2 inhibitors increased ultrafiltration potentially through the maintenance of the glucose gradient upon the inhibition of SGLT2 activity in the peritoneum, although conflicting results exist [7, 9, 10]. In addition, SGLT2 inhibitors were reported to mitigate peritoneal fibrosis and angiogenesis, both of which contribute to ultrafiltration failure, in a mouse model [8, 11]. Based on this information, we hypothesized that SGLT2 inhibitors might increase the ultrafiltration volume and improve long-term prognosis in patients on PD by correcting volume overload and mitigating peritoneal tissue damage. Because there is little information on the use of SGLT2 inhibitors in patients on PD, we decided to first evaluate the effect of SGLT2 inhibitors on the ultrafiltration volume in short-term interventions.

## Methods

### Study design

The EMPOWERED trial is a multicenter, randomized, double-blind, placebo-controlled, crossover trial evaluating the efficacy and safety of once-daily oral empagliflozin 10 mg in patients on PD. This trial has been registered at the Japan Registry of Clinical Trials (jRCTs051230081). The trial was approved by the Osaka University Clinical Research Review Committee (approval number: S23004), and it is being conducted according to the 1964 Declaration of Helsinki and its later amendments or comparable ethical standards. This trial will be conducted at four academic and community hospitals in Japan (i.e., Osaka University Hospital, National Cerebral and Cardiovascular Center, Osaka General Medical Center, and Matsuyama Red Cross Hospital). Written, informed consent will be obtained from all individual participants included in the study. The trial is being managed by the Academic Clinical Research Center of Osaka University Hospital with the cooperation of intellim Corporation, a Contract Research Organization, which is responsible for monitoring, data management, and statistical analysis. This trial received funds from Nippon Boehringer Ingelheim Co., Ltd. and Eli Lilly Japan K.K. under a research agreement with Osaka University. Boehringer Ingelheim Pharma GmbH & Co. KG is providing drugs for the study. This study was collaboratively designed with input from both academic members and representatives from Nippon Boehringer Ingelheim. Patient enrollment will be started in December 2023, and the scheduled completion date is October 2024.

### Study participants

Enrolled participants must meet all inclusion criteria and none of the exclusion criteria listed in Table 1. Key inclusion criteria include an age of 18–90 years, PD vintage ≥ 3 months, use of ≥ 3 L/day glucose-based PD solution, and a diagnosis of and treatment for chronic heart failure. The Japanese Ministry of Health, Labor, and Welfare had approved empagliflozin for the treatment of type 2 diabetes and chronic heart failure at the time of manuscript submission. However, because of a lack of efficacy in the hypoglycemic effect, empagliflozin is not approved for type 2 diabetes in patients on dialysis in Japan. Therefore, inclusion criteria for chronic heart failure were established to conduct the study under the approved indications in Japan. These include elevated N-terminal pro-brain natriuretic peptide (NT-proBNP)/brain natriuretic peptide (BNP) levels, structural heart disease, elevated left ventricular filling pressure, or a history of hospitalization for heart failure. The definitions of structural heart disease and elevated filling pressure are presented in Supplementary Table 1. Key exclusion criteria include treatment with SGLT2 inhibitors within 3 months of enrollment, hybrid therapy with PD and hemodialysis, and peritonitis within 2 months of enrollment. These criteria were selected because rapid ultrafiltration by hemodialysis and peritonitis can alter peritoneal function.

**Table 1:** Inclusion and exclusion criteria

Inclusion criteria	Age ≥ 18 and ≤ 90 years BNP ≥ 40 pg/mL, NT-proBNP ≥ 400 pg/mL, structural heart disease (left atrial enlargement and/or left ventricular hypertrophy), elevated filling pressures, or a history of hospitalization for heart failure* Standard medical therapy for heart failure (at least one of the following: loop diuretics, ACEIs, ARBs, ARNIs, beta-blockers, or MRAs) PD vintage ≥ 3 months Glucose-based PD solution use ≥ 3 L/day Voluntarily participate with written informed consent
Exclusion criteria	Treatment with SGLT2 inhibitors within 3 months before enrollment Individuals who are not expected to survive more than 1 year after enrollment On a hybrid therapy comprising peritoneal dialysis and hemodialysis Individuals who have or have had peritonitis within the past 2 months Women who are pregnant or nursing Active infections Individuals who participate in clinical studies (trials and research) involving other interventions Individuals disqualified from participation in the study by the investigator or sub-investigator for any other reasons

*NT-proBNP must be used to confirm eligibility for participants receiving ARNIs

*BNP* brain natriuretic peptide; *NT-proBNP* N-terminal pro-brain natriuretic peptide; *ACEIs* angiotensin-converting enzyme inhibitors; *ARBs* angiotensin receptor blockers; *ARNIs* angiotensin receptor neprilysin inhibitors; *MRAs* mineralocorticoid receptor antagonists; *PD* peritoneal dialysis; *SGLT2* Sodium-Glucose Cotransporter 2

### Randomization

The participants will be randomized to the empagliflozin or placebo group at a 1:1 ratio. This allocation will be executed utilizing a computer-generated random sequence and will be stratified by study sites, using a permuted block randomization method. Because the study is double-blinded, patients, their families, the study team, and the sponsor will not have access to the randomization information until the trial database is locked.

### Intervention periods

**Fig. 1:**
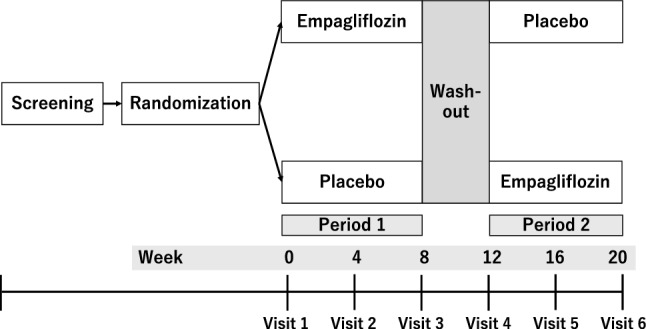
Study scheme. Participants will be randomized to receive empagliflozin 10 mg once daily and then placebo or vice versa. Each treatment period will last 8 weeks, with a 4-week washout period in between. After randomization, patients will visit every 4 weeks during the study period

Figure 1 displays a scheme for this trial. The study includes a two-period crossover consisting of 8 weeks of treatment and a 4-week washout between treatment periods. Participants will visit every 4 weeks and continue on trial medication, namely empagliflozin 10 mg or placebo, once daily, in each period. The utilization of SGLT2 inhibitors, excluding empagliflozin, is strictly proscribed, and changes in PD prescriptions are fundamentally prohibited. Adherence to the study drugs will be monitored by interviewing the patients at every visit and counting tablets at the end of each period. Table 2 presents the observation and evaluation schedule during the study period.

**Table 2:**
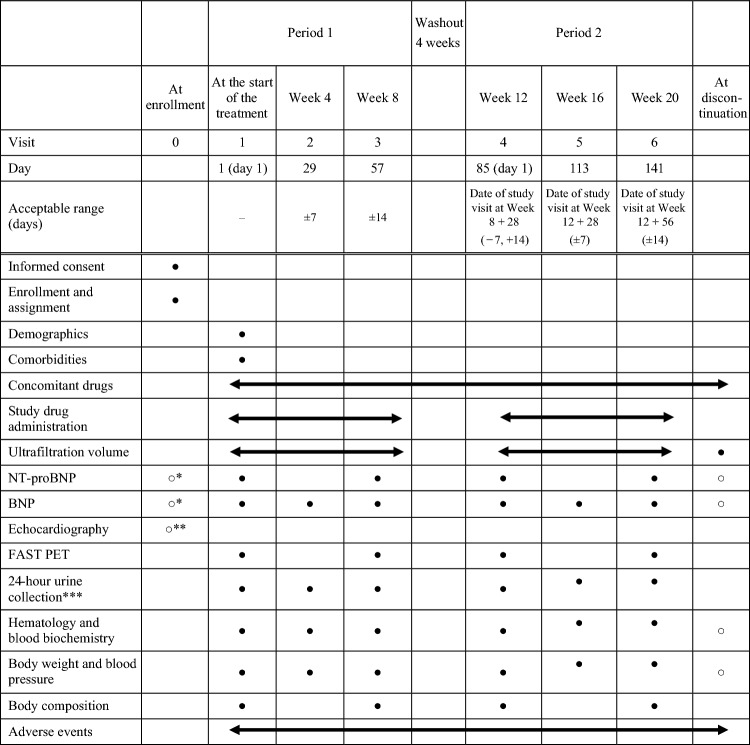
Observation, test, and evaluation schedule

*NT-proBNP*, N-terminal pro-brain natriuretic peptide; *BNP* brain natriuretic peptide; *FAST PET* frequently and short time peritoneal equilibration test; *KIM-1* kidney injury molecule 1

●, required; ○, if necessary

*Data collected from routine medical practice 182 days before providing informed consent may be used for assessment

**Data collected from routine medical practice 365 days before providing informed consent may be used for assessment

***Urine KIM-1 is measured only on Visit 3 and Visit 6

### Primary endpoint

The primary endpoint of the study is the change in the daily ultrafiltration volume from baseline to week 8 in each intervention period. The ultrafiltration volume from non-glucose-based PD solution (i.e., icodextrin-containing PD solution) will be excluded. The daily ultrafiltration volume will be calculated as the mean of all results for 5 of 7 consecutive days, after excluding the maximum and minimum values. The participants will record data for each session including the type of PD solution, dwell time, and instilled/drained volume of the PD solution.

### Secondary endpoints

Secondary outcomes are changes from baseline to week 8 in each intervention period as follows: (1) NT-proBNP and BNP levels; (2) the levels of biomarkers related to frequently and short time peritoneal equilibration test (FAST PET) [12] (ultrafiltration volume, sodium, potassium, glucose, urea nitrogen, creatinine, uric acid, protein, interleukin-6, cancer antigen 125, and drained solution-to-serum creatinine ratio); (3) the levels of biomarkers related to 24-h urine collection (urine volume, sodium, potassium, glucose, urea nitrogen, creatinine, uric acid, urine protein, and urea and creatinine clearance); (4) the levels of anemia-related factors (hemoglobin, hematocrit, ferritin, and transferrin saturation); (5) body weight, blood pressure and, body composition (intracellular and extracellular fluid volume). Details of FAST PET are shown in Supplementary Table 2. Urine kidney injury molecule 1 (KIM-1) levels will be compared at week 8 in each intervention period. Adverse events will be also evaluated during the study period.

### Sample size calculation

Our preliminary data revealed an average increase in the daily ultrafiltration volume from the glucose-based PD solution of 90 mL/day in five patients on PD who received 10 mg of empagliflozin and used at least 3 L/day glucose-based PD solution. An increase in the ultrafiltration volume of 90 mL/day is associated with an 18% reduction in the risk of death [4], which appears to be clinically significant. Based on the Osaka University cohort of patients on PD, the between-subject standard deviation (SD) of the change in the ultrafiltration volume was conservatively estimated at 150 mL (actual between-subject SD of the cohort was 114 mL), and the ratio of the between-subject SD to the within-subject SD was 1:1. We calculated that 30 patients completing the study would provide 90% power (at α = 0.05, two-sided) to detect a 90 mL difference in the ultrafiltration volume between empagliflozin and placebo. The sample size was set at 36 subjects to compensate for potential dropouts.

### Statistical analysis

Two different analysis sets, namely the full and safety analysis sets (FAS and SAS, respectively), will be used in the study. The FAS will include all study participants who receive at least one dose of the study drug and complete at least one post-baseline examination or assessment. The SAS will include all the study participants who receive at least one dose of the study drug. Analyses of the primary and secondary endpoints excluding adverse events will be conducted in the FAS. Adverse events will be compared between the two groups in the SAS. To assess the effects of empagliflozin versus placebo on the primary outcome, we will use a mixed-effects model for repeated measures (MMRM). The model includes treatment, period, and treatment-by-period interaction as fixed effects and individual as random effects. An unstructured marginal covariance structure will be specified. Secondary endpoints for continuous variables will be estimated in the same manner as described for primary endpoints excluding urine KIM-1. In the case of urinary KIM-1, the analysis using MMRM will be conducted with the absolute value at the end of each period as the dependent variable. Skewed data will be log-transformed before analyses. A list of adverse events will be summarized by tables for each treatment. For sensitivity analyses, we will employ a similar analysis by excluding the data with catheter dysfunction (i.e., drainage volume < 80% of the instilled volume or taking ≥ 20 min to instill the PD solution). A subgroup analysis will be performed using the following factors: age (< 65 years/ ≥ 65 years), sex, presence or absence of diabetes, use of icodextrin-containing PD solution, 24-h urine volume (< 200 mL/ ≥ 200 mL), the glucose load from the peritoneal dialysate (concentration × volume per day, median), the glucose concentration of the drained PD solution on FAST PET (median), and the drained PD solution-to-serum creatinine ratio (median) on FAST PET. Missing data will not be imputed. Statistical analyses will be performed using SAS software (SAS Institute, Cary, NC, USA). Interim analysis will not be conducted.

## Discussion

A large amount of evidence supports the use of SGLT2 inhibitors in individuals with heart failure or chronic kidney disease (CKD) [13, 14]. However, few studies have examined treatment with SGLT2 inhibitors in patients with advanced CKD, especially those requiring dialysis. Because SGLT2 is expressed somewhat specifically in renal tubules [15], it is theoretically possible that the effectiveness of SGLT2 inhibitors could diminish as renal function worsens. On the contrary, emerging data indicate that the cardiovascular and kidney benefits of SGLT2 inhibitors do not wane even as the estimated glomerular filtration rate (eGFR) declines at least in patients with eGFR ≥ 20 mL/min/1.73 m^2^ [16, 17]. Furthermore, in the DAPA-CKD study (trial registration: NCT03036150), in which patients continued taking the study drug after starting renal replacement therapy, dapagliflozin achieved a 21% relative risk reduction in mortality within the subset of patients who commenced dialysis therapy (unpublished data described in the rationale for the RENAL LIFECYCLE Trial [18]). These observations raise the possibility that SGLT2 inhibitors can exert their organ-protective effects through mechanisms distinct from merely blocking SGLT2 in renal tubules.

The current trial was prompted by a report in 2019 that SGLT2 is expressed in the human peritoneum [7]. If SGLT2 plays a role in glucose absorption from the PD solution, SGLT2 inhibitors might enhance the ultrafiltration volume by preserving the glucose gradient across the peritoneum membrane. Although animal studies have yielded inconsistent results [7, 9, 10], recent human case series reported the positive effects of SGLT2 inhibitors on the ultrafiltration volume [19, 20]. Therefore, we planned to conduct a clinical trial evaluating the impact of SGLT2 inhibitors on the ultrafiltration volume. To the best of our knowledge, only two other small randomized, controlled trials (RCT) are currently investigating the effects of SGLT2 inhibitors in patients on PD. One of these trials is an open-label RCT involving 36 patients with type 2 diabetes on PD assessing BNP as the primary endpoint (trial registration: jRCT1011210022). The other trial is a double-blind, placebo-controlled, crossover RCT with 30 patients on PD, and the primary endpoint is glucose absorption from the PD solution (trial registration: NCT05671991). Notably, our trial is particularly unique in that the primary endpoint is the daily ultrafiltration volume, which is an important indicator for patients on PD [3, 4], and the impact on residual renal function will be evaluated. These ongoing RCTs are expected to furnish robust evidence regarding the efficacy and safety of SGLT2 inhibitors in patients on PD.

## Conclusion

The EMPOWERED trial will evaluate the efficacy and safety of empagliflozin in patients on PD. The results of this study could reveal the beneficial effect of SGLT2 inhibitors for fluid management in this specific population.

### Supplementary Information

Below is the link to the electronic supplementary material.

Supplementary file1 (DOCX 26 KB)

## Data Availability

Data from this study will be made available upon reasonable request after consultation with the chief investigator and the sponsor.

## References

[1] Cho Y, Bello AK, Levin A, Lunney M, Osman MA, Ye F (2021). Peritoneal dialysis use and practice patterns: an international survey study. Am J Kidney Dis.

[2] Rhee H, Baek MJ, Chung HC, Park JM, Jung WJ, Park SM (2016). Extracellular volume expansion and the preservation of residual renal function in Korean peritoneal dialysis patients: a long-term follow up study. Clin Exp Nephrol.

[3] Ateş K, Nergizoğlu G, Keven K, Sen A, Kutlay S, Ertürk S (2001). Effect of fluid and sodium removal on mortality in peritoneal dialysis patients. Kidney Int.

[4] Lin X, Lin A, Ni Z, Yao Q, Zhang W, Yan Y (2010). Daily peritoneal ultrafiltration predicts patient and technique survival in anuric peritoneal dialysis patients. Nephrol Dial Transplant.

[5] Teitelbaum I (2021). Peritoneal dialysis. N Engl J Med.

[6] Kim YL, Biesen WV (2017). Fluid overload in peritoneal dialysis patients. Semin Nephrol.

[7] Zhou Y, Fan J, Zheng C, Yin P, Wu H, Li X (2019). SGLT-2 inhibitors reduce glucose absorption from peritoneal dialysis solution by suppressing the activity of SGLT-2. Biomed pharmacother Biomed Pharmacother.

[8] Balzer MS, Rong S, Nordlohne J, Zemtsovski JD, Schmidt S, Stapel B (2020). SGLT2 inhibition by intraperitoneal dapagliflozin mitigates peritoneal fibrosis and ultrafiltration failure in a mouse model of chronic peritoneal exposure to high-glucose dialysate. Biomolecules.

[9] Schricker S, Oberacker T, Fritz P, Ketteler M, Alscher MD, Schanz M (2022). Peritoneal expression of SGLT-2, GLUT1, and GLUT3 in peritoneal dialysis patients. Kidney Blood Press Res.

[10] Martus G, Bergling K, de Arteaga J, Öberg CM (2021). SGLT2 inhibition does not reduce glucose absorption during experimental peritoneal dialysis. Perit Dial Int.

[11] Shentu Y, Li Y, Xie S, Jiang H, Sun S, Lin R (2021). Empagliflozin, a sodium glucose cotransporter-2 inhibitor, ameliorates peritoneal fibrosis via suppressing TGF-β/Smad signaling. Int Immunopharmacol.

[12] Twardowski ZJ (1990). The fast peritoneal equilibration test. Semin Dial.

[13] Heidenreich PA, Bozkurt B, Aguilar D, Allen LA, Byun JJ, Colvin MM (2022). 2022 AHA/ACC/HFSA guideline for the management of heart failure: executive summary: a report of the American college of cardiology/American heart association joint committee on clinical practice guidelines. J Am Coll Cardiol.

[14] de Boer IH, Khunti K, Sadusky T, Tuttle KR, Neumiller JJ, Rhee CM (2022). Diabetes management in chronic kidney disease: a consensus report by the American diabetes association (ADA) and kidney disease: improving global outcomes (KDIGO). Kidney Int.

[15] Santer R, Calado J (2010). Familial renal glucosuria and SGLT2: from a mendelian trait to a therapeutic target. Clin J Am Soc Nephrol CJASN.

[16] Zannad F, Ferreira JP, Pocock SJ, Zeller C, Anker SD, Butler J (2021). Cardiac and kidney benefits of empagliflozin in heart failure across the spectrum of kidney function: insights from EMPEROR-reduced. Circulation.

[17] Herrington WG, Staplin N, Wanner C, Green JB, Hauske SJ, Emberson JR (2023). Empagliflozin in patients with chronic kidney disease. N Engl J Med.

[18] The RENAL LIFECYCLE Trial: A RCT to assess the effect of dapagliflozin on renal and cardiovascular outcomes in patients with severe CKD - full text view - ClinicalTrials.gov. 2023. https://clinicaltrials.gov/ct2/show/NCT05374291.

[19] Alhwiesh AK, Abdul-Rahman IS, Nasreldin MA, Mohammed AM, Al-Oudah S, Al-Thwainy R (2022). The use of SGLT2 inhibitors in peritoneal dialysis patients: a shade of light on dapagliflozin. Arch Nephrol Urol.

[20] Lai JW, Lin HJ, Chou CY (2023). SGLT-2 inhibitors may increase ultrafiltration in incident peritoneal dialysis patients: a case report. BMC Nephrol.

