# Supplementary material for: Effects of sodium-glucose co-transporter 2 inhibitors on ultrafiltration in patients with peritoneal dialysis: a protocol for a randomized, double-blind, placebo-controlled, crossover trial (EMPOWERED)
Source: Clin Exp Nephrol. 2024 Feb 25;28(7):629–35. doi: 10.1007/s10157-024-02467-w (PMC11189947; doi:10.1007/s10157-024-02467-w)
Supplement: Supplementary file 1 — Supplementary file1 (DOCX 26 KB) [file 10157_2024_2467_MOESM1_ESM.docx]

Supplementary Table 1. Definition of structural heart disease (left atrial enlargement/left ventricular hypertrophy) and ventricular filling pressure*

| Left atrial enlargement | LA width ≥4.0 cm, or  LA length ≥5.0 cm, or  LA area ≥20 cm^2^, or  LA volume (LAV) ≥55 mL, or  LA volume index (LAVI) ≥34 mL/m^2^ |
| --- | --- |
| Left ventricular hypertrophy | Interventricular septal thickness (IVST) or left ventricular posterior wall thickness (LVPWth) ≥1.1 cm, or  LV mass index (LVMI) ≥115 g/m^2^ (men) and ≥95 g/m^2^ (women) |
| Ventricular filling pressure | E/e′ (mean septal and lateral) ≥13, or  e′ (mean septal and lateral) <9 cm/s |

LA, left atrial

*Cited and modified from Anker SD, Butler J, Filippatos G, et al. Empagliflozin in Heart Failure with a Preserved Ejection Fraction. N Engl J Med. 2021 Oct 14;385(16):1451-1461.

Supplementary Table 2. Details in Frequently and Short-time Peritoneal Equilibration Test*

| Step |  |
| --- | --- |
| 1 | Before commencing the procedure, ensure all windows and doors are closed, fans are switched off and dusting and cleaning is kept to a minimum. |
| 2 | Connect patient to warm 2 liter of peritoneal dialysate solutions (or equivalent osmotic dialysate) equivalent to 2.27% glucose concentration. |
| 3 | Drain out PD effluent from patient for at least 20 minutes to ensure the peritoneum is completely empty and ensure accuracy of the test. |
| 4 | Perform a 15 second flush before fill procedure, |
| 5 | Infuse the 2litre peritoneal dialysate solutions into the patient. |
| 6 | Note the exact time after all the dialysate is infused and time a 4 hour dwell. |
| 7 | At four hours, connect patient to appropriate peritoneal dialysate bag. |
| 8 | Drain out effluent fluid from patient for at least 20 minutes to ensure peritoneum completely empty and to ensure accuracy of the test. |
| 9 | At four hours, take a blood sample from the patient. |
| 10 | Perform 15 second flush and complete peritneal dialysis exchange in accordance with local standard operation procedure. |
| 11 | Draw out effluent samples from drainage bag as follows:  a) Gently shake the bag of PD effluent to mix the contents.  b) Wipe the medication port with a 2% Chlorhexidine swab (or equivalent) and allow to dry.  c) Using an aseptic non touch technique, with a 10ml sterile syringe and sterile needle, draw a 10ml sample of effluent from the medication port. |
| 12 | Document procedure performed in nursing notes with record of volume of effluent drained in and out. |

*Cited and modified from Morelle J, Stachowska-Pietka J, Öberg C, et al. ISPD recommendations for the evaluation of peritoneal membrane dysfunction in adults: Classification, measurement, interpretation and rationale for intervention. Perit Dial Int. 2021 Jul;41(4):352-372.
